# Maternal Health Literacy Progression Among Rural Perinatal Women

**DOI:** 10.1007/s10995-014-1432-0

**Published:** 2014-01-28

**Authors:** Sandra C. Mobley, Suzanne Dixson Thomas, Donald E. Sutherland, Jodi Hudgins, Brittany L. Ange, Maribeth H. Johnson

**Affiliations:** 1 Department of Obstetrics and Gynecology, Georgia Regents University, Augusta, GA USA; 2 SW Campus, Medical College of Georgia, Georgia Regents University, Augusta, GA USA; 3 Enterprise Community Healthy Start, Department of Ambulatory Care, Georgia Regents University, Augusta, GA 30912 USA; 4 CSRA Nursing Associates, PC, 300 Gardners Mill Court, Augusta, GA 30907 USA; 5 Evaluation Services, Medical College of Georgia, Georgia Regents University, Augusta, GA 30912 USA; 6 Department of Biostatistics and Epidemiology, Medical College of Georgia, Georgia Regents University, Augusta, GA 30912-4900 USA

**Keywords:** Health literacy, Healthy Start, Home visitation, Infant mortality, Intermediate factors, Life Skills Progression Instrument, Maternal health literacy, Perinatal case management

## Abstract

This research examined changes in maternal health literacy progression among 106 low income, high risk, rural perinatal African American and White women who received home visits by Registered Nurse Case Managers through the Enterprise Community Healthy Start Program. Maternal health literacy progression would enable women to better address intermediate factors in their lives that impacted birth outcomes, and ultimately infant mortality (Lu and Halfon in Mater Child Health J 7(1):13–30, 2003; Sharma et al. in J Natl Med Assoc 86(11):857–860, 1994). The Life Skills Progression Instrument (LSP) (Wollesen and Peifer, in Life skills progression. An outcome and intervention planning instrument for use with families at risk. Paul H. Brookes Publishing Co., Baltimore, 2006) measured changes in behaviors that represented intermediate factors in birth outcomes. Maternal Health Care Literacy (LSP/M-HCL) was a woman’s use of information, critical thinking and health care services; Maternal Self Care Literacy (LSP/M-SCL) was a woman’s management of personal and child health at home (Smith and Moore in Health literacy and depression in the context of home visitation. Mater Child Health J,  2011). Adequacy was set at a score of (≥4). Among 106 women in the study initial scores were inadequate (<4) on LSP/M-HCL (83 %), and on LSP/M-SCL (30 %). Significant positive changes were noted in maternal health literacy progression from the initial prenatal assessment to the first (*p* < .01) postpartum assessment and to the final (*p* < .01) postpartum assessment using McNemar’s test of gain scores. Numeric comparison of first and last gain scores indicated women’s scores progressed (LSP/M-HCL; *p* < .0001) and (LSP/M-SCL; *p* < .0001). Elevated depression scores were most frequent among women with <4 LSP/M-HCL and/or <4 LSP/M-SCL. Visit notes indicated lack or loss of relationship with the father of the baby and intimate partner discord contributed to higher depression scores.

## Introduction

### Purpose and Setting

This research examined changes in maternal health literacy progression among low income, high risk, rural, African American (AA) and White women who received home visits by Registered Nurse Case Managers (RNCMs) through the Enterprise Community Healthy Start (ECHS). Perinatal case management encompassed prenatal through up to 24 months postpartum care. ECHS served two rural Georgia counties with health professional shortages in all categories [5] and with no public transportation. Georgia’s state health outcomes ranked 37th nationally, and study counties’ outcomes ranked 156th and 144th of 159 [6]. ECHS’s caseload included over 80 % AA women. In 2010, AA infant mortality in Georgia’s East Central Health District which included ECHS counties was four times (16.4 IMR) that of Whites (4.0 IMR) [7]. ECHS served women with high medical, social, economic, and/or psychological risks. This report documents changes in maternal health literacy progression of women enrolled in their first experience of perinatal case management in the Enterprise Community Healthy Start Program.

### Scientific and Theoretical Background

Persistently high infant mortality in the US led to an intense focus upon socio-bio-behavioral determinants of health [1, 2, 8–10]. National goals specified reduction of low birth weight, premature or immature delivery, fetal death, and unplanned or unwanted pregnancies, especially among teens [10–14]. Lowering infant mortality could not be accomplished with medical care alone [8, 15]. Perinatal care was re-focused as a broad community-driven public health intervention designed to reach women and infants outside clinical settings spanning preconceptional through interconceptional care and home visiting [16–20]. Home visiting had long term benefits in life skills development [21–28] and had demonstrated effectiveness in Healthy Start programs [29–34]. Case management was essential to empower women to address intermediate risk and protective factors in their lives through improved maternal health literacy that impacted birth outcomes and infant survival [35–39].

*Maternal Health Literacy (MHL)* meant women employed cognitive and social skills based in experience to access, understand and evaluate information to promote health for themselves and their children [37–41]. Initially, health literacy focused upon reading and numeracy skills [42–45] then shifted to functional health literacy [37]; the shift was critical for serving women with low educational levels [32–36]. Smith and Moore [4] found that women were able increase their MHL despite perinatal depression. Because perinatal depression impacted infant survival [1, 46–50], it was included in this study to evaluate its potential impact upon MHL progression.

## Methods

### Design

The design was a retrospective analysis of existing records for women served by ECHS, with pre-post comparison of prenatal to initial and to final postpartum; internal comparison groups structured analysis of secondary outcomes [51, 52]. A post hoc review of visit notes clarified women’s circumstances that might have modified outcomes observed.

### Study Sample

The study included women having their first experience of case management, who were admitted to case management after July 1, 2005, and who had had at least one prenatal and one postpartum LSP assessment with a live birth. Of 611 women, 106 met study criteria. Subject selection was cut off February 1, 2010, and by March 31, 2012 all subjects had completed case management. Progression was measured using two clusters of LSP items relevant to perinatal care [4]. Individual item and composite item cluster scores provided outcome data. Smith’s and Moore’s multi-state sample of over 5000 included 32 ECHS women who also were included as subjects in the present study [4]. Their LSP initial prenatal scores were proportionately distributed across the internal comparison study groups in this study.

*Registered Nurse Case Managers (RNCMs)* were four AA women experienced in perinatal care. Each RNCM provided care for the same women across time for continuity of care. RNCMs taught women from *Beginnings Pregnancy Guide* [53, 54] and other resources about their health, their babies, parenting, and how to use the health care system.

## Data Collection and Measurement

### Instrumentation

*The Life Skills Progression Instrument* (LSP) was an important advancement in measurement of MHL progression [3]. Two clusters of LSP items were established to measure MHL (Table 1). LSP/Health Care Literacy (LSP/M-HCL) was the mother’s ability to obtain, critically evaluate, and apply information that would enable her to utilize the health care system for herself and her child. LSP/Self Care Literacy (LSP/M-SCL) was the mother’s ability to care for herself and her child at home [4]. Content validity was established with input from multiethnic expert reviewers. Wollesen developed guidelines for use of the LSP with the *Think*-*Link*-*Respond* process of reflective functioning that promoted changes and formulated interpersonal bonds with their fetus/infant [55–60]. Guidelines were linked to the *Beginnings Pregnancy* *Guide* curriculum [54]. LSP validity measurements were α = .64 to .99; inter-rater reliability was estimated at 90 % (3). The LSP items were not defined as scales. Items used were selected for relevance to perinatal care and demonstrated content validity with RNCMs in the study [56, 57, 61].

**Table 1:** Maternal health care literacy and maternal self care literacy items

Item #/label	Description	Factors used by home visitor to determine score
*Maternal Health Care Literacy Items (LSP/M-HCL)*		
10. Use of information f = 368	Degree to which accepts and uses information from home visitor and other reliable sources in health care	Self-report during interaction with Registered Nurse Care Manager (RNCM) Completed referral appointments
17. Prenatal Care f = 143	The initiation and follow through with consistently keeping provider visits Scored only during pregnancy	Self-report from client Review of provider’s records
18. Parent Sick Care f = 367	Degree to which seeks and uses medical home appropriately, as well as follows treatment plan	Self-report during interaction with RNCM RNCM’s check of medications remaining
19. Family planning f = 269	Use and understanding of method; spacing pregnancies	Self-report during interaction with RNCM Reproductive health planning process Hospital and physician records
20. Child Well Care f = 259	Medical home use for well child care Scored only during postpartum LSPs	Maternal self-report during interaction with RNCM Physician records
21. Child Sick Care f = 248	Degree to which seeks and uses medical home appropriately and in a timely manner for child, as well as follows treatment plan Scored only during postpartum	Self-report during interaction with Registered Nurse Care Manager (RNCM) RNCM’s observation of client and child Hospital and physician records
22. Child Dental Care f = 66 “No Teeth”	Dental home use and degree of treatment and hygiene Scored only for infants age 6–12 months	Self-report during interaction with RNCM RNCM’s observation of client and child
23. Child immunizations f = 259	Ranges from refusal to degree of completion Scored only for infants age 6–12 months	Self-report during interaction with RNCM Immunization registry Physician records
33. Health insurance f = 330	None (1) through private insurance (5)	Self-report during interaction with RNCM Hospital records
*Maternal Self Care Literacy Items (LSP/M-SCL)*		
4. Attitudes toward pregnancy f = 124	Attitudes ranged from unplanned and unwanted to planned, prepared, and welcomed. Scored only on prenatal LSPs	RNCM’s observation of client’s comments about pregnancy, emotions displayed and actions re: preparation for baby
7. Support of development f = 260	Knowledge, interest, and involvement in promoting development of child Scored only on postpartum LSPs	RNCM’s observation of client and child Ages and Stages Questionnaire (ASQ) [74] completion
8. Safety f = 269	Home/car/environment safe for child; treatment of unintentional injury; seeks and uses information Scored only on postpartum LSPs	RNCM’s observation of client and child Hospital records
11. Use of resources f = 367	Degree to which woman identified and used community resources independently, and kept or rescheduled appointments	Honored appointments with RNCM or called if there was a conflict Percentage of kept referral appointments Self-report of resources independently used
24. Substance use or abuse f = 368	History and use/abuse of drugs and/or alcohol	HV observation of client and family Self-report during interaction with RNCM Knowledge of resources and use of referrals for treatment for severe problems
25. Tobacco f = 370	Level of smoking and exposure to second-hand smoke	HV observation of client and family Self-report during interaction with RNCM
28. Self Esteem f = 372	Covers range from self-critical and lacking initiative to confident and verbalizes pride in successes	Self-report during interaction with RNCM

Frequency *(f)* was the number of scores from a possible total of 373 LSP assessments for 106 women. All women had at least one prenatal assessment and one postpartum assessment. Infant dental care was not assessed until age ≥6 months; others were assessed at age-appropriate times

Both the *Edinburgh Postpartum Depression Survey* (EPDS) [62] and the *Beck Depression Inventory* (BDI) [63] had been validated for depression screening in perinatal populations, including AAs. Use of both instruments enabled staff to confirm screening results and measure two dimensions of depression during the perinatal period [46–49]. The EPDS, a ten-item self-report scale, was developed in 1987 for use in postpartum populations in community samples. It had both depression and anxiety subscales, focused on mood aspects of depression, did not include somatic items, and was sensitive to intervention response. The EPDS had a maximum score of 30. A score of 10 or more might indicate possible depression of varying severity. The BDI, a 21-item self-report scale, with a range of 0–63, contained psychic and somatic items; a score of 13 or greater was considered positive for depression. Measurement of somatic items might reflect normal physiologic symptoms of pregnancy [51]. In a meta-analysis of 30 studies of perinatal depression, both the BDI and the EPDS had acceptable sensitivity and high specificity for either major depression or postpartum depression or both. We set scores for BDI at ≥13 or EPDS at ≥10 as “screened positive” for evidence of perinatal depression [46].

### Generation of Scores

RNCMs completed the first LSP assessment approximately 2 months from admission and after home visits and some office and community contacts, which permitted the RNCM time to establish a relationship with the client and collaborate on a plan of care. Postpartum LSPs were performed ideally at 2 months postpartum and every 6 months thereafter. Scores on LSP items consisted of a 0–5 rating in .5 increments on each item that pertained to the woman’s perinatal stage. Zero indicated not applicable, no answer, or not asked; one (1) meant very inadequate and five (5) meant competent. RNCMs scored women’s behaviors on the basis of their repeated interactions with the women in their caseloads.

Women completed depression scale items during case management visits that were then scored by RNCMs, who referred women with elevated scores; but local non-emergent mental health services were extremely limited. The EPDS [62] and BDI [63] were administered prenatally at 34–36 gestational weeks; and after delivery at 3–6 weeks; then at 6 month intervals.

### Data Record

RNCMs recorded data in the ECHS electronic Perinatal Database. Final data for the study were extracted for analysis after March 31, 2012. To prevent scoring bias, women’s LSP scores were not tied to personnel evaluations. RNCMs were encouraged to use their professional nursing judgment as they assessed women’s responses. Data were reviewed weekly and monthly for completeness and accuracy.

### Demographic Data Used for Analyses

Demographic data were recorded at entry into case management, including: maternal age, race, years of education, insurance status, gravidity, and marital status. Data were adjusted for age-appropriate educational achievement. Birth outcomes were infant’s gestational age, birth weight, neonatal intensive care unit (NICU) admissions, and presence of congenital defects. *ELF* was a measure of literacy as reading and numeracy skills among low income patients (65). We used a combination score of age-appropriate education (*E*) (yes = 2, no = 1), father of the baby (FOB) lives in the home (*L*) (yes = 2, no = 1) and mother of baby reads for fun (*F*) (yes = 2, no = 1). ELF scores ranged from 3 to 6.

### Assignment Method: Internal Comparison Groups

Final LSP/M-HCL and LSP/M-SCL scores provided a basis for comparison among women to examine factors that might further explicate results. The only criteria for group assignment were final LSP overall scores on the two clusters of items. Women who ended case management with scores less than four (<4) for the LSP/M-HCL cluster were assigned to Group I; the LSP/M-SCL cluster, Group II; or both LSP/M-HCL and LSP/M-SCL, Group III. Women who had final LSP scores greater than or equal to four (≥4) for both clusters were assigned to Group IV [53, 66].

### Blinding Method

RNCMs and women in the study were not made aware of the study. Clients were not told of their LSP assessments. Investigators had no direct input into case management or LSP scoring. It was difficult for RNCMs to look at past LSP scores.

### Units of Analysis

The smallest units of analysis were a woman’s LSP/M-HCL and M-PHL scores on an LSP assessment. Initial and final postpartum LSP/M-HCL and LSP/M-SCL scores were treated as outcome scores to be compared to the initial prenatal LSP/M-HCL and LSP/M-SCL scores.

### Analysis

SAS© version 9.3 (SAS Institute, Inc., Cary, NC) was used for all analyses. The level of significance was set at .05 for all tests.

## Hypotheses

High risk perinatal women will demonstrate MHL progression during case management as evidenced by differences between the first prenatal LSP/M-HCL and LSP/M-SCL scores compared to the final postpartum LSP/HCL and LSP/SCL scores.

High risk perinatal women will demonstrate MHL progression during case management as evidenced by differences between the first prenatal LSP/M-HCL and LSP/M-SCL scores compared to the first postpartum LSP/HCL and LSP/SCL scores.

### Primary Outcome Analyses

We examined criterion-based (±4) evidence of changes in LSP/M-HCL and LSP/M-SCL scores from the first prenatal to first postpartum and to final postpartum LSP assessments for the 106 women in the study. McNemar’s test was used to evaluate the paired data. Paired odds ratios (OR) and 95 % confidence intervals (CI) were calculated. Progression to adequacy was calculated as an odds ratio for change from inadequate (<4) LSP/M-HCL and LSP/M-SCL scores to adequate or competent (≥4).

Numeric gain scores (positive, negative or zero) for final postpartum compared to baseline prenatal LSP assessments served as numeric indices of MHL progression. Numeric scores were calculated without regard to the standard of 4, since progression might be noted as a change from a very low score of 1.0 to a score of 3.9 without meeting the criterion of adequacy. Overall gain scores were calculated for LSP/M-HCL and LSP/M-SCL separately. Paired Student’s *t* tests were used to evaluate means of numeric gain scores. Gain scores for LSP/M-HCL and LSP/M-SCL were tested for correlation using Pearson’s r.

### Secondary Outcome Analyses

Baseline comparisons were made for those who scored < 4 and those who scored ≥4 on the LSP/M-HCL and LSP/M-SCL prenatal LSP assessments. Pooled (equal variances) or Satterthwaite (unequal variances) *t* tests were used for continuous variables and Chi square tests were used for categorical variables.

### Visit Notes Reviewed

Visit notes were reviewed for the 37 low-scoring women to examine factors that might help to explain their outcomes.

### Formation of Internal Comparison Groups

Final postpartum LSP/M-HCL and LSP/M-SCL scores were treated as outcome scores. A score of 4 or greater was deemed adequate. We observed an internal division among subjects’ final postpartum LSP scores that enabled us to make a comparative evaluation of the impact of perinatal case management by factors that may be social determinants of perinatal health [1, 2]. Women who ended case management with <4 outcome scores for the LSP/M-HCL cluster of items formed Group I; <4 on the LSP/M-SCL cluster of items, Group II; <4 on LSP/M-HCL and LSP/M-SCL, Group III. Women who ended case management with ≥4 for the LSP/M-HCL and the LSP/M-SCL clusters of items formed Group IV, the adequate/competent group.

Groups I, II, and III (n = 37) were combined to evaluate demographic and personal factors that may have contributed to lower (<4) outcome scores on LSP/M-HCL and/or LSP/M-SCL when compared with Group IV (n = 69). Pooled (equal variances) or Satterwaite (unequal variances) *t* tests were used for continuous variables and Chi square tests were used for categorical variables.

## Results

### Primary Outcome

Support was found for the main hypotheses of the study; nulls were rejected

Tests of the study’s hypotheses demonstrated significant positive changes to adequate scores (≥4) on the LSP/HCL and LSP/SCL clusters of items from the initial LSP assessment to the final LSP assessment at the end of case management. The women were 57 times more likely to score as adequate on the final postpartum LSP/HCL (*p* < .0001, 95 % CI (8, 403), S = 54.1) and 3.0 times as likely to score as adequate on the final postpartum LSP/SCL (*p* = .0082, 95 % CI (1.3, 7.1), S = 7.0) than the prenatal assessments. The women were 13.3 times more likely to score as adequate on the first postpartum LSP/HCL (*p* < .0001, 95 % CI (4.8, 36.6), S = 42.1) and 2.8 times as likely to score as adequate on the first postpartum LSP/SCL (*p* = .011, 95 % CI (1.2, 6.2), S = 6.5) than the prenatal assessments.

In a first-to-last numeric comparison of LSP/M-HCL gain scores without regard to the criterion for adequacy, a paired Student’s *t* test indicated significant positive gains from prenatal LSP/M-HCL to final postpartum LSP/M-HCL scores (t = 13.0, n = 106, *p* < .0001); the mean (±SD) change was .59 (±.47). In a first-to-last numeric comparison of LSP/M-SCL gain scores without regard to the criterion for adequacy, a paired Student’s *t* test indicated significant positive gains from prenatal LSP/M-SCL to final postpartum LSP/M-SCL scores (t = 4.7, n = 106, *p* < .0001); the mean (±SD) change was .23 (±.49).

Women’s LSP/M-HCL and LSP/M-SCL gain scores were moderately correlated (Pearson’s r = .47). Visual inspection yielded no clear pattern of factors on which LSP/HCL scores regressed. Among women whose scores regressed in LSP/M-SCL, scores dropped in “attitude toward pregnancy,” “support of infant’s development,” and “self-esteem.”

#### Instrument

Scoring frequencies are shown in Table 1. Item 17 Prenatal Care of the LSP/M-HCL cluster has 5 score positions: 1 through 4 relate to a woman’s prenatal care. Position 5 related to whether or not she had a postpartum medical visit. Frequencies were low for item 17. All women in the study received a score on Item 17 on their prenatal LSP assessments. RNCMs evidenced confusion about how to score the postpartum visit or when to score if it was beyond eight weeks. When the RNCMs did score the item in a postpartum LSP, the added scores did not change the woman’s overall score (i.e., above or below 4), nor her group position (I-IV), with four exceptions: the four women’s final scores changed from 3 to 4. Removal of all item 17 data from the analyses increased scores on the LSP. To prevent score inflation, we retained all scores on Item 17.

Item 3 of the LSP/M-HCL cluster was a measure of an infant’s access to dental care. LSP instructions were not to score the item until the infant had teeth. Scores also reflected limited access to dental services due to lack of providers who accepted Medicaid-insured infants and lack of public transportation in either county.

### Secondary Outcomes

#### Baseline Before Case Management

At the baseline prenatal LSP assessment, 88 women had <4 LSP/M-HCL scores and 18 women had ≥4 LSP/M-HCL scores. Demographic factors tested were age (adult vs. teen) (*p* = .02); race (Black vs. Other) (*p* = .69); gravidity (multiparous vs. primaparous) (*p* = .25); and education in years (<12 vs. ≥12) (*p* = .03). Mean years of education (<12 vs. ≥12) (*p* = .02) and age (*p* = .02) were statistically significantly different. A greater proportion of women who scored ≥4 at baseline were teens (n = 12, 77.8 %) than those who scored <4 (n = 32, 36.4 %). Women who scored ≥4 LSP/M-HCL at baseline had higher average number of years of education (12.8 ± 3.1) than did those who scored <4 LSP/M-HCL at baseline (11.0 ± 1.9). Similarly, a greater proportion of women scoring ≥ 4 had at least 12 years of education (n = 14, 44.8 %).

At the baseline prenatal LSP assessment, 32 women had <4 LSP/M-SCL scores and 74 women had ≥4 LSP/M-SCL scores. The Combined Group I, II, II and Group IV women were not significantly different (*p* < .05) in age (adult vs. teen), race (Black vs. Other), or gravidity (multiparous vs. primaparous). Mean years of education (*p* = .0003) and years of education (<12 vs. ≥ 12) (*p* = .008) were statistically significantly different. Women who scored adequate or competent (≥4) LSP/M-SCL at baseline had higher average number of years of education (11.8 ± 2.2) than did those who scored less than adequate (<4) LSP/M-SCL at baseline (10.2 ± 1.9). Similarly, a greater proportion of women scoring ≥ 4 had at least 12 years of education (n-46, 62.2 %) compared to women who scored <4 (n = 11, 34.4 %).

#### Outcomes After Case Management

Outcomes enabled investigators to form internal comparison groups by which we examined factors that might further inform the results and guide future case management (Table 2). Women with adequate LSP outcomes were older, had more years of education, were less likely to be on Medicaid, and were more likely to read for fun than those who did not have adequate final LSP scores on both assessments.

**Table 2:** Demographic characteristics of women in the study by outcome groups

Demographic	Group I	Group II	Group III	Combined Group I, II, III	Group IV	Tests of significance α = .05
	Low LSP/M-HCL	Low LSP/M-SCL	Low LSP/M-HCL and LSP/M-SCL	Lowest	Highest	Combined Group I-II-III versus Group IV
	LSP/M-HCL <4 (n = 18)	LSP/M-SCL <4 (n = 8)	LSP/M-HCL <4 and LSP/M-SCL <4 (n = 11)	LSP/M-HCL and/or LSP/M-SCL <4 (n = 37)	LSP/M-HCL and LSP/M-SCL ≥4 (n = 69)	
Age, year	22 ± 4	19 ± 3	20 ± 7	21 ± 5	23 ± 7	0.47^b^
Age						.882^c^
Teens	4 (22)	4 (50)	7 (64)	15 (41)	29 (42)	
Adults	14 (78)	4 (50)	4 (36)	22 (59)	40 (58)	
Education, years	11 (±2)	10 (±2)	10 (±2)	11 (±2)	12 (±2)	.018^a^
Age-appropriate educational level						.663^c^
Yes	11 (61)	4 (50)	7 (64)	22 (59)	58 (84)	
No	7 (39)	4 (50)	4 (36)	15 (41)	11 (16)	
Insurance status						.099^c^
Medicaid	16 (89)	8 (100)	10 (91)	34 (92)	59 (84)	
Other	0 (0)	0 (0)	0 (0)	0 (0)	7 (10)	
None	2 (11)	0 (0)	1 (9)	3 (8)	4 (6)	
Gravidity	2 ± 6	1.8 ± 1.5	1.5 ± .7	1.8 ± 1.4	2 ± 1.4	.502^a^
Marital status						**1.0^c^ (single vs. other)
Single	13 (7)	7 (88)	10 (91)	30 (81)	53 (77)	
Separated, divorced or widowed	2 (11)	0 (0)	1 (9)	3 (8)	7 (10)	
Married	3 (17)	1 (12)	0 (0)	4 (11)	9 (13)	
FOB lives in home						.003^c^
Yes	12 (67)	3 (37.5)	3 (27)	18 (49)	53 (77)	
No	6 (33)	5 (62.5)	8 (73)	19 (51)	16 (23)	
ELF Scores, median	4	4	3	4	5	
Race						.293^c^ (AA vs. Other)
African American	13 (72)	6 (75)	9 (82)	28 (76)	58 (84)	
Caucasian	5 (28)	2 (25)	2 (18)	9 (24)	10 (14)	
All others (mixed)	0 (0)	0 (0)	0 (0)	0 (0)	1 (1)	

^a^Pooled *t* test, mean ± SD

^b^Satterthwaite *t* test, mean ± SD

^c^Chi Square test, n (column  %)

#### Depression in the Internal Comparison Groups

Using a paired *t* test, we compared mean depression scores prenatal to postpartum of the Combined Group (I, II, and III) with Group IV (Table 3). BDI scores dropped following delivery for the Combined Group (*p* = .002) and Group IV (*p* = .0003); but EPDS scores did not decline significantly for either the Combined Group (*p* = .069) or for Group IV (*p* = .32). Antepartum EPDS mean scores were higher in the Combined Group than in Group IV (*p* = .0004); but the mean antepartum BDI score in the Combined Group was not significantly different from the mean antepartum BDI score in Group IV (*p* = .054) again signifying differences in the two sets of depression measures. Thus, women who had higher antepartum depression scores did not have adequate MHL on the prenatal LSP assessment of LSP/M-HCL and LSP/M-SCL; but depression scores following delivery did not explain differences in outcome LSP/M-HCL and LSP/M-SCL scores.

**Table 3:** Prenatal and postpartum depression scores for women in the study (N = 106) by outcome groups

Prenatal and postpartum depression scores	Group I	Group II	Group III	Combined Group I, II, III	Group IV	Tests of significance α = .05
	Low LSP/M-HCL	Low LSP/M-SCL	Low LSP/M-HCL and LSP/M-SCL	Lowest	Highest	Combined Group I, II, III compared to Group IV
	LSP/M-HCL <4 (n = 18)	LSP/M-SCL <4 (n = 8)	LSP/M-HCL <4 and LSP/M-SCL <4 (n = 11)	LSP/M-HCL and/or LSP/M-SCL <4 (n = 37)	LSP/M-HCL and LSP/M-SCL ≥4 (n = 69)	
Antepartum EPDS scores	8.9 ± 5.4	9 ± 9.1	15.2 ± 4.2	10.9 ± 6.5	6.6 ± 5.2	.0004^a^
Low (<10)	10 (56)	6 (75)	1 (9)	17 (46)	54 (78)	.0007^c^
High (≥10)	8 (44)	2 (25)	10 (91)	20 (54)	15 (22)	
Postpartum EPDS scores	5.4 ± 5.1	7.9 ± 3.3	12.8 ± 9.1	8.1 ± 6.8	5.7 ± 6.4	.086^a^
Low (<10)	16 (89)	5 (62)	5 (45)	26 (70)	56 (81)	.202^c^
High (≥10)	2 (11)	3 (38)	6 (55)	11 (30)	13 (19)	
Prenatal BDI scores	10.2 ± 7.3	11.9 ± 7.3	18.5 ± 4.1	13.1 ± 7.5	9.7 ± 8.6	.054^a^
Low (<13)	13 (72)	5 (62)	1 (9)	19 (51)	54 (78)	.004^c^
High (≥13)	5 (28)	3 (38)	10 (91)	18 (49)	15 (22)	
Postpartum BDI scores	5.2 ± 3.8	8.9 ± 6.2	12.7 ± 8.0	8.2 ± 6.5	6.3 ± 6.7	.183^a^
Low (<13)	17 (94)	7 (88)	6 (55)	30 (81)	60 (87)	.420^c^
High (≥13)	1 (6)	1 (12)	5 (45)	7 (19)	9 (13)	

^a^Pooled *t* test, mean ± SD

^b^Satterthwaite *t* test, mean ± SD

^c^Chi Square test, n (column  %)

Birth Outcomes are shown in Table 4. Greater length of time in case management was an important factor in MHL progression. Of 106 women in the study group 37 (34 %) completed 24 months of postpartum case management. Of the 21 who became pregnant again, the median number of months from delivery to conception was 11.25 (range 1.64 to 20.07). Eighty-nine percent of women in the Combined Group had fewer than 24 months of postpartum case management compared to 62 percent in Group IV (*p* = .0034). Women in Group IV had more LSP assessments (*p* < .001).

**Table 4:** Birth outcomes for women in the study (N = 106) by outcome groups

Birth outcomes	Group I	Group II	Group III	Combined Group I, II, III	Group IV	Tests of significance α = .05
	Low LSP/M-HCL	Low LSP/M-SCL	Low LSP/M-HCL and LSP/M-SCL	Lowest	Highest	Combined Group I, II, III compared to Group IV
	LSP/M-HCL <4 (n = 18)	LSP/M-SCL <4 (n = 8)	LSP/M-HCL <4 and LSP/M-SCL <4 (n = 11)	LSP/M-HCL and/or LSP/M-SCL <4 (n = 37)	LSP/M-HCL and LSP/M-SCL ≥4 (n = 69)	
Gestational age, week	38.7 ± 2.4	38.8 ± 1.9	38.4 ± 2.1	38.6 ± 2.2	38.1 ± 2.1	.252^a^
Birth weight, g	3,237 ± 633	2,966 ± 545	2,960 ± 370	3,096 ± 552	3,101 ± 631	.964^a^
≥2,500 g	17 (94)	7 (88)	9 (82)	33 (89)	58 (84)	
1,500–2,499 g	0 (0)	1 (12)	2 (18)	3 (8)	10 (14)	
<1,500 g	1 (6)	0 (0)	0 (0)	1 (3)	1 (2)	
NICU Admissions	1 (6)	0 (0)	0 (0)	1 (3)	2 (3)	.793^c^
Infants with congenital anomaly	1 (6)	0 (0)	0 (0)	1 (3)	2 (3)	.954^c^

^a^Pooled *t* test, mean ± SD

^b^Satterthwaite *t* test, mean ± SD

^c^Chi Square test, n (column %)

Visit notes revealed low-scoring women had problems with housing, intimate partner discord/loss, lack of personal/public transportation, return to work, and early end to case management.

## Discussion, Limitations and Conclusions

Years of education was important in women’s LSP/M-HCL and LSP/M-SCL baseline prenatal scores [66, 67] but final outcomes reflected MHL progression through case management [7] Gravidity was not related to outcome scores, suggesting that the ECHS program of perinatal case management was a greater contributor to women’s maternal literacy progression [31–36]. Length of case management was important to women’s success. Health information, parenting skills and reflective functioning may have made it possible for most of the women in the study group to progress [52–57]. Scoring objectivity was evidenced in score fluctuations, regressions, and the number of women who did not reach adequacy (≥4).

Evidence of perinatal depression as measured by the EPDS was different from evidence of chronic depression as measured by the BDI. Because of their high risk circumstances, it is likely depression was a chronic underlying problem. Visit notes revealed intimate partner discord was important in perinatal depression [47, 64, 65, 68, 69]. Further study of family structure and paternal involvement in mother and infant care was needed [71, 72]. Depression was a deterrent to women’s initial success, but despite their depression women’s MHL increased [4]. Attrition reflected difficulties in communication, tracking, and social circumstances. Further studies were indicated to follow up the 21 women who became pregnant before 24 months. Further examination of dosage factors may reveal additional information about the impact of perinatal case management upon MHL progression [70].

The LSP was an important breakthrough in documenting a dynamic, complex nurse-patient relationship in a community setting with home visiting as an important venue for service delivery. The LSP addressed critical issues in maternal child health care. An item for preconceptional and interconceptional health could be added to the LSP [73]. Item 22 (oral health care) should be updated to reflect current practice. LSP data made possible analyses on nominal through numeric scales. Further information about the LSP’s sensitivity and specificity was needed to refine measurements. Ages and Stages (ASQ-3) data may be useful in further analyses of the impact of perinatal case management upon children's development [74]. Using the internal group design for secondary analyses and visit notes revealed important information about intermediate factors that may have influenced MHL progression. The long term stable relationship with RNCMs supported women’s progress in MHL.

## References

[1] Lu MC, Halfon N (2003). Racial and ethnic disparities in birth outcomes: A life-course perspective. Maternal Child Health Journal.

[2] Sharma R, Synkewcz C, Raggio T, Mattison DR (1994). Intermediate variables as determinants of adverse pregnancy outcome in high-risk inner-city populations. Journal of the National Medical Association.

[3] Wollesen L, Peifer K (2006). Life skills progression. An outcome and intervention planning instrument for use with families at risk.

[4] Smith, S, & Moore, E. J. (2011). Health literacy and depression in the context of home visitation. *Maternal and Child Health Journal*. doi 10.1007/210995-011-0920-8. 10.1007/s10995-011-0920-8 PMC3443343 22120425

[5] US Department of Health and Human Services (USDHHS). (2012). Health Resource Services Administration (HRSA). Health Professional Shortage Areas (HPSA) [cited 12-1-2012]. http://www.hrsa.gov/hpsa.

[6] Forum One Communications, University of Wisconsin Population Health Institute, Robert Wood Johnson Foundation, and Burness Communications. (2012). County health rankings and roadmaps [cited 3-25-13]. www.countyhealthrankings.org/health-outcomes.

[7] Georgia Department of Community Health. Online Analytical Statistical Information (OASIS). (2012). Maternal and infant statistics [cited 10-1-2012]. http://www.oasis.state.ga.us.

[8] Devaney, B, Howell, E, McCormick, M, & Moreno, L. (2000). Reducing infant mortality. Lessons learned from Healthy Start. Princeton, NJ: Mathematica Policy Research, Inc. (submitted to DHHS BPHC HRSA. Contract No.:240-93-0050; MPR Ref No 8166-113).

[9] Moreno L, Devaney B, Chu D, Seeley M (2000). Effect of Healthy Start on infant mortality and birth outcomes.

[10] USDHHS Centers for Disease Control and Prevention (CDC). (2013). Infant mortality among Black Americans. Morbidity and Mortality Weekly Report. 1-16-1987; 36(1):1–4, 9–10 [cited 1-17-2013]. http://www.cdc.gov/mmwr/preview/mmwrhtml/000000850.htm. 3099157

[11] USDHHS CDC National Center for Health Statistics. Infant mortality statistics from the 2008 period linked birth/infant death data set. (2012). National Vital Statistics Reports, 60(5):1–27. 24974588

[12] Betancourt JR, King (2003). Unequal treatment: The Institute of Medicine Report and its public health implications. Public Health Reports.

[13] USDHHS. Healthy People. (2010). Maternal, infant and child health objectives [cited 10-1-2012]. http://www.healthypeople.gov.

[14] USDHHS. Healthy People. (2020). Maternal, infant and child health objectives [cited 10-1-2012]. http://www.healthypeople.gov.

[15] Fine, A, & Kotelchuk, M. (2010). Rethinking MCH: The life course model as an organizing framework. Concept paper. In *Proceedings of the USDHHS HRSA MCH, 75th Anniversary Conference October*, Rockville, MD, pp. 1–18.

[16] Rosenbach M, O’Neill S, Cook B, Trebino L, Walker DK (2010). Characteristics, access, utilization, satisfaction and outcomes of Healthy Start participants in eight sites. Maternal and Child Health Journal.

[17] Halfon N, DuPlessis H, Inkelas M (2007). Transforming the US child health system. Health Affairs.

[18] Halfon, N, & Hochstein, M. (2002). Life course health development: an integrated framework for developing health policy and research. *The Milbank Quarterly, 80*(3), 1–28 [cited 9-21-2010]. http://milbank.org/quarterly. 10.1111/1468-0009.00019 PMC2690118 12233246

[19] USDHHS Maternal and Child Health. (2011). Improving maternal health care: The next generation of research. (Conference date 9-(18-19)-2000) [cited 4-27-2011]. http://www.ahrq.gov/research/maternhlth/mathealth1.htm.

[20] Howell, E. (2000). *Effect of Healthy Start on infant mortality and birth outcomes*. Princeton NJ: Mathematica Policy Research, Inc. (submitted to DHHS Bureau of Primary Health Care (BPHC) HRSA. Contract No.:240-93-0050; MPR Ref No 8166-111).

[21] Hench, K. (2012). Healthy start. Promising practices. DHHS BPHC HRSA 2008 [cited 11-12-2012]. https://www.promisingpractices.net/programs.

[22] Olds DL, Henderson CR, Tatelbaum R, Chamberlin R (1988). Improving the life course development of socially disadvantaged mothers: A randomized trial of nurse home visitation. American Journal of Public Health.

[23] Echenrode J, Campa M, Luckey DW, Henderson CR, Cole R, Kitzman H, Anson E, Sidora-Arcoleo K, Powers J, Olds D (2010). Long-term effects of prenatal and infancy nurse home visitation on the life course of youths. Archives of Pediatrics and Adolescent Medicine.

[24] Temple P, Lutenbacher M, Vitale J (2008). Limited access to care and home healthcare. Clinical Obstetrics and Gynecology.

[25] Byrd ME (1997). A typology of the potential outcomes of maternal-child home visits: A literature analysis. Public Health Nursing.

[26] Byrd ME (1998). Long-term maternal-child home visiting. Public Health Nursing.

[27] Hanks CS, Smith J (2002). Implementing nurse home visitation programs. Public Health Nursing.

[28] McNaughton DB (2004). Nurse home visits to maternal-child clients: A review of intervention research. Public Health Nursing.

[29] Margolis PA, Stevens R, Bordley WC, Stuart J, Harlan C, Keyes-Elstein L, Wisseh S (2001). From concept to application: The impact of a community-wide intervention to improve the delivery of preventive services to children. Pediatrics.

[30] USDHHS Healthy Start. (2012). Home visiting. Home visiting evidence of effectiveness [cited 11-10-12]. https://www.homevee.act.hhs.gov.

[31] Salihu HM, Mbah AK, Jeffers D, Alio AP, Berry L (2009). Healthy start program and feto-infant morbidity outcomes: evaluation of program effectiveness. Maternal and Child Health Journal.

[32] Will JA, Hall I, Cheney T, Driscoll M (2005). Flower power: Assessing the impact of the Magnolia Project on reducing poor birth outcomes in an at-risk neighborhood. Journal of Applied Sociology/Sociological Practice.

[33] Stabile, I, & Graham, M. (2000). *Florida Panhandle Healthy Start: A randomized trial of prenatal home visitation*. Tallahassee, FL: Florida State University Center for Prevention and Early Intervention Policy [cited 1-15-2013]. http://eric.ed.gov/contentdelivery/servlet/ERICServlet?accno=ED444747.

[34] McCormick MC, Deal LW, Devaney BL, Chu D, Moreno L, Raykovich KT (2001). The impact on clients of a community-based infant mortality reduction program: The National Healthy Start Program Survey o Postpartum Women. American Journal of Public Health.

[35] Nabukera, S. K., Wingate, M. S., Owen, J., Salihu, H. M., Swaminathan, S., Alexander, G. R., & Kirby, R. S. (2009). Racial disparities in perinatal outcomes and pregnancy spacing among women delaying initiation of childbearing. *Maternal and Child Health Journal, 3*(1), 81–89 [cited 3-9-2010]. http://medscape.com/viewarticle/586708. 10.1007/s10995-008-0330-8 18317891

[36] Wells N, Sbrocco T, Hsiao CW, Hill LD, Vaughn NA, Lockley B (2008). The impact of nurse case management home visitation on birth outcomes in African-American women. Journal of the National Medical Association.

[37] Nutbeam D (2000). Health literacy as a public health goal: A challenge for contemporary health education and communication strategies into the 21st century. Health Promotion International.

[38] Renkert K, Nutbeam D (2001). Opportunities to improve maternal health literacy through antenatal education: An exploratory study. Health Promotion International.

[39] Nutbeam D (2002). Health literacy: A search for new categories. Health Promotion International.

[40] Smith, B. J., Tang, K. C., & Nutbeam, D. (2006). WHO health promotion glossary: New terms. *Health Promotion International, 21*(4), 340. doi:10.1093/heapro/da1033. 10.1093/heapro/dal033 16963461

[41] Nutbeam D (1999). The challenge to provide ‘evidence’ in health promotion. Health Promotion International.

[42] Low Health Literacy Linked to Higher Risk of Death and More Emergency Room Visits and Hospitalizations. (2011 March). Agency for Healthcare Research and Quality, Rockville, MD. http://www.ahrq.gov/news/newsroom/press-releases/2011/lowhlit.html.

[43] Bennett IM, Robbins S, Haecker T (2003). Screening for low literacy among adult caregivers of pediatric patients. Family Medicine.

[44] USDHHS. (2012). National action plan to improve health literacy [cited 10-16-2012]. http://www.health.gov/communication/HLActionPlan/.

[45] USDHHS AHRQ. (2011). Health literacy interventions and outcomes: An updated systematic review. March 2011 [cited 10-16-2012]. http://www.ahrq.gov/clinic/tp/lituptp.htm.

[46] Gaynes, B. N., Gavin, N., Meltzer-Brody, S., Lohr, K. N., Swinson, T., Gartlehner, G. et al. (2005). Perinatal depression: Prevalence, screening accuracy, and screening outcomes. Evidence report/technology assessment no. 119. (Prepared by the RTI-University of North Carolina Evidence-based Practice Center, under Contract No. 290-02-0016.) Agency for Health Care Research and Quality (AHRQ) Pub. No. 05-E006-2. February 2005.

[47] Smith MV, Shao L, Howell H, Lin H, Yonkers KA (2011). Perinatal depression and birth outcomes in a Healthy Start project. Maternal and Child Health Journal.

[48] Luke S, Salihu H, Alio A, Mbah A, Jeffers D, Lo Berry E (2009). Risk factors for major antenatal depression among low-income African American women. Journal of Women’s Health.

[49] Straub, H., Adams, M., Kim, J. J., & Silver, R. K. (2012). Antenatal depressive symptoms increase the likelihood of preterm birth [cited 11-10-12]. *American Journal of Obstetrics & Gynecology,* 207, x-ex/ex; 1.e1–1.e4. http://www.AJOG.org. 10.1016/j.ajog.2012.06.033 22789523

[50] Shadish WR, Cook TD, Campbell DT (2002). Experimental and quasi-experimental designs for generalized causal inference.

[51] Coyle, S. L., & Boruch, R. F. (1991). *Turner, CF. Evaluating AIDS Prevention Programs. National Research Council (US). Panel on the evaluation of AIDS interventions*. Washington, DC: National Academy Press. 25144077

[52] Smith S (2005). Beginnings pregnancy guide.

[53] Smith, S. A. (2010). Home visits using reflective approach improve functional health literacy among low-income pregnant women and new parents [cited 10-19-2010]. http://innovations.ahrq.gov/content.aspx?id=2533.

[54] Smith S, Wollesen L (2004). Beginnings life skill development curriculum: Home visitor handbook.

[55] Wollesen L (2005). On-site training; Enterprise Community Healthy Start (ECHS), Perinatal Center.

[56] Wollesen L (2010). On-site training; ECHS, Perinatal Center.

[57] Wollesen, L. (2008). Reflective function. An exercise in collaborative thinking #1. Presented at on-site training for ECHS, Perinatal Center, Georgia Regents University, Augusta, GA, 2008.

[58] Slade, A., Sadler, L. S., & Mayes, L. C. (2005). Minding the baby: Enhancing parental reflective functioning in a nursing/mental health home visiting program. In L. J. Berlin, Y. Ziv, L. Amaya-Jackson, & M. T. Greenberg (Eds), *Enhancing early attachments. Theory, research, intervention and policy. Duke series in child development and public policy* (Vol. xxiv, pp. 152–177). New York: Guildford Press.

[59] Grienenberger J, Kelly K, Slade A (2005). Maternal reflective functioning, mother-infant affective communication, and infant attachment. Attachment and Human Development.

[60] Brinberg D, McGrath JE (1985). Validity and the research process.

[61] Cox JL, Holden JM, Sagovsky R (1987). Detection of postnatal depression. Development of the Edinburgh Postnatal Depression Scale (EPDS). British Journal of Psychiatry.

[62] Beck AT (1961). An inventory for measuring depression. Archives of General Psychiatry.

[63] Hobfoll SE, Ritter C, Lavin J, Hulsizer MF, Cameron RP (1995). Depression prevalence and incidence among inner-city pregnant and postpartum women. Journal of Consulting and Clinical Psychology.

[64] Sit DKY, Flint C, Svidergol D, White J, Wimer M, Bish B, Wisner KL (2009). An emerging best practice model for perinatal depression care. Psychiatric Services.

[65] O’Mahen HA, Flynn HA (2008). Preferences and perceived barriers to treatment for depression during the perinatal period. Journal of Women’s Health.

[66] Ohnishi M, Nakamura K, Takano T (2005). Improvement in maternal health literacy among pregnant women who did not complete compulsory education: Policy implications for community care services. Health Policy.

[67] Weiss BD, Francis L, Senf JH, Heist K, Hargraves R (2006). Literacy education as a treatment for depression in patients with limited literacy and depression. Journal of General Internal Medicine.

[68] Wadhwa PD, Entringer S, Buss C, Lu MC (2011). The contribution of maternal stress to preterm birth: Issues and considerations. Clinics in Perinatology.

[69] Scribano, P. V., Stevens, J., & Kaizar, E. (2012). The effects of intimate partner violence before, during, and after pregnancy in nurse visited first time mothers. *Maternal and Child Health Journal*. doi:10.1007/s10995-.12-0986-y. 10.1007/s10995-012-0986-y 22426619

[70] Slaughter JC, Issel LM (2012). Developing a measure of prenatal case management dosage. Maternal and Child Health Journal.

[71] Alio AP, Bond MJ, Padilla YC, Heidelbaugh JJ, Lu M, Parker WJ (2011). Addressing policy barriers to paternal involvement during pregnancy. Maternal and Child Health Journal.

[72] Fouquier KF (2011). The concept of motherhood among three generations of African American women. Journal of Nursing Scholarship.

[73] Posner, S. F., Johnson, K., Parker, C., Atrash, H., & Biermann, J. (2006). The national summit on preconception care: A summary of concepts and recommendations. *Maternal and Child Health Journal,**10*, S197–S205. doi:10.1007/s10995-006-0107-x. 10.1007/s10995-006-0107-x PMC1592248 16773451

[74] Squires, J. & Bricker, D. (2009). *Ages & Stages Questionnaires (ASQ-3): A parent-completed child-monitoring system* (3rd ed.). Baltimore, MD: Brookes Publishing Co.

